# MNX1 Promotes Malignant Progression of Cervical Cancer via Repressing the Transcription of p21^cip1^

**DOI:** 10.3389/fonc.2020.01307

**Published:** 2020-08-11

**Authors:** Biqing Zhu, Yaqin Wu, Jing Luo, Quanli Zhang, Jian Huang, Qian Li, Lin Xu, Emei Lu, Binhui Ren

**Affiliations:** ^1^Department of Radiation Oncology, Jiangsu Cancer Hospital, Jiangsu Institute of Cancer Research, Nanjing Medical University Affiliated Cancer Hospital, Nanjing, China; ^2^Department of Cardiothoracic Surgery, Jinling Hospital, Medical School of Nanjing University, Nanjing, China; ^3^Jiangsu Key Laboratory of Molecular and Translational Cancer Research, Nanjing, China; ^4^Department of Thoracic Surgery, Jiangsu Cancer Hospital, Jiangsu Institute of Cancer Research, Nanjing Medical University Affiliated Cancer Hospital, Nanjing, China

**Keywords:** cervical cancer, MNX1, cell cycle, transcription, p21^cip1^

## Abstract

Motor neuron and pancreas homeobox 1 (MNX1) is a development-related genes and has been found to be highly expressed in several cancers. However, its biological function in cervical cancer remains largely unexplored. QRT-PCR, western blot, and IHC showed that MNX1 was abnormally overexpressed in cervical cancer tissues and cell lines. The high expression level of MNX1 correlated with poorer clinicopathologic characteristics in cervical cancer patients. Evaluated by RTCA (Real Time Cellular Analysis) proliferation assay, colony formation assay, EdU assay, transwell assay, and matrigel assay, we found that knockdown of MNX1 inhibited proliferation, migration and invasion of cervical cancer *in vitro*, while overexpression of MNX1 promoted malignant phenotype of cervical cancer. And subcutaneous xenograft model confirmed the malignant phenotype of MNX1 *in vivo*. Furthermore, flow cytometry, chromatin immunoprecipitation, and luciferase reporter assay indicated that MNX1 accelerated cell cycle transition by transcriptionally downregulating cyclin-dependent kinases p21^cip1^. In summary, our study revealed that MNX1 exerted an oncogenic role in cervical cancer via repressing the transcription of p21^cip1^ and thus accelerating cell cycle progression. Our results suggested that MNX1 was a potential diagnostic marker and therapeutic target for cervical cancer patients.

## Introduction

As one of the most common gynecological malignant tumors, cervical cancer is the fourth leading cause of cancer-related death among women worldwide (1). Although efforts (including periodic cancer screening, prompt surgical treatment, chemotherapy, and radiotherapy) have been made to decrease the mortality of cervical cancer, the prognosis of patients is still poor and cervical cancer remains an important public health issue (2). The pathogenesis of cervical cancer has not been clearly illustrated, but it is confirmed that the activation of tumor-promoting genes and the inactivation of tumor suppressor genes participate in the progression of cervical cancer (3). To screen for novel abnormally expressed genes functioning in cervical cancer may provide potential prognostic markers and therapeutic targets for treatment.

MNX1 (Motor neuron and pancreas homeobox 1, also known as HB9, HLXB9) is a member of homeobox gene family and encodes a nuclear protein (4). The homeobox genes are a group of genes containing homeobox (a 180 base pairs long DNA sequence) and encode homeodomain proteins that act as transcription factors (5). Many homeobox genes have been proved to be implicated in various human cancers, acting as oncogenes, or tumor suppressors (6–8). MNX1 was firstly found to be expressed in lymphoid and pancreatic tissues and defined as a novel human homeobox gene in 1994 (9). Early studies showed that MNX1 was involved invertebrate and pancreatic development (10, 11) and motor neuronal differentiation (12). Defects in this gene result in Currarino syndrome, an autosomic dominant congenital malformation (13). In follow-up study, MNX1 was found to be abnormally expressed in several cancer types, including prostate cancer, hepatocellular carcinoma and acute myeloid leukemia (14–16). Furthermore, recent studies confirmed that MNX1 played oncogenic roles in colorectal cancer, breast cancer, and bladder cancer (17–19).

The aim of this study is to identify the expression and function of MNX1 in cervical cancer. Our results revealed that MNX1 was significantly upregulated in cervical cancer and correlated with poorer prognosis. The knockdown or overexpressed MNX1 inhibited or promoted aggressiveness of cervical cancer, including proliferation, migration, and invasion capacities, by enhancing or repressing the transcription of p21^cip1^ thus regulating the G2/M cell cycle transition. These findings suggested that MNX1 might be a potential diagnostic marker and therapeutic target for cervical cancer.

## Materials and Methods

### Bioinformatics

The TCGA dataset termed TCGA_CESC_exp_HiS-eqV2-2015-02-24 was downloaded from the UCSC cancer browser (https://genome-cancer.ucsc.edu/) (20) to evaluate the expression of MNX1 in cervical cancer and adjacent normal tissues. GEPIA (Gene Expression Profiling Interactive Analysis) (http://gepia.cancer-pku. cn/index.html) was used to analyze the expression of MNX1 with Disease Free Survival (DFS) of cervical cancer patients. The cBioPortal website (http://www. cbioportal.org/) (21) was utilized to obtain highly co-expressed genes with MNX1. Totally 208 genes highly correlated with MNX1 (Pearson score > 0.4; Table S1) were submitted to DAVID Bioinformatics Resources 6.8 (http://david.abcc.ncifcrf.gov/) (22) for Gene Ontology (GO), Kyoto Encyclopedia of Genes and Genomes (KEGG), and Reactome Pathway analysis. And we analyzed the binding site of MNX1 and p21^cip^ promoters through the Jaspar Database (http://jaspardev.genereg.net/) (23).

### Human Cervical Cancer Cell Lines

The human normal cervical cell lines (Hacat) and cervical cancer cell lines (HeLa, Siha, Caski, and C33a) were purchased from American Type Culture Collection (ATCC, USA). HeLa, Siha, C33a, and Hacat cells were incubated in DMEM medium (KeyGEN, Nanjing, China), and Caski cells were cultured in RPMI1640 (KeyGEN, Nanjing, China) medium containing 10% fetal bovine serum (GIBCO-BRL, Invitrogen, Carlsbad, CA, USA) and cultured at 37°C in a humidified incubator containing 5% CO_2_.

### Human Cervical Cancer Tissues

The 40 pairs of cervical cancer tissues and adjacent tissues were selected from the Affiliated Cancer Hospital of Nanjing Medical University and informed consent was obtained from all subjects. All tumors and paired non-tumor tissues were confirmed by experienced pathologists and no patients received chemotherapy or radiotherapy before surgery. The mRNA expression of MNX1 and p21^cip1^ in cervical cancer tissues was detected by qRT-PCR. Collection of human tissue samples was conducted in accordance with the International Ethical Guidelines for Biomedical Research Involving Human Subjects (CIOMS). This study was approved by the Ethics Committee of the Nanjing Medical University Affiliated Cancer Hospital.

### Tissue Microarrays

Paired cervical cancer tissue microarrays were obtained from Shanghai Outdo Biotech Co., Ltd. (Cat. No. OD-CT-RpUtr03-004 and OD-CT-RpUtr03-006). Totally 62 pairs of paraffin-embedded human cervical cancer sections were analyzed for MNX1 expression. All tissues were examined by two experienced pathologists and the TNM stage was confirmed in each patient with blinded methods. The sections were incubated with an anti-MNX1 primary antibody (1:100, Abcam, ab79541). The IHC scores were calculated according to intensity and percentage of positive cells. The staining intensity was evaluated as the basis of four grades: 0 (negative staining), 1(weak staining), 2 (moderate staining), or 3 (strong staining). The product (percentage of positive cells and respective intensity scores) was used as the final staining scores (a minimum value of 0 and a maximum value of 300).

### RNA Preparation, Reverse Transcription, and qRT-PCR

TRIzol reagent (Invitrogen, Carlsbad, CA, USA) was used to extract total RNA from tissue samples or cultured cells according to the manufacturer's protocol. A Reverse Transcription Kit (Takara, Cat: RR036A, KeyGEN) was utilized to generate cDNA. QRT-PCR was performed with SYBR Select Master Mix (Applied Biosystems,Cat: 4472908. KeyGEN, Nanjing, China) and primers are shown in Table S2.

### Western Blotting

Lysis buffer (RIPA, KeyGEN) containing protease inhibitors (PMSF, KeyGEN) was used to extract protein of cells and tissues, and protein concentration was detected with a BCA Kit (KeyGEN). Protein samples (40 μg) were loaded into 10% sodium dodecyl sulfate polyacrylamide electrophoresis (SDSPAGE) gels and transferred onto a PVDF membrane after electrophoresis. The membrane was blocked with non-fat milk for 2 h, and incubated overnight with antibodies against respective antibodies: MNX1 (Abcam, ab79541, 1:1,000); p21^cip1^ (Cell Signaling Technology; 2947, 1:1,000); p^Thr161^-CDK1 (Cell Signaling Technology, 9114, 1:1,000); CDK1 (Cell Signaling Technology, 9116, 1:1,000); p27^kip1^ (Cell Signaling Technology, 3686; 1:1,000); CyclinB1 (Abcam, ab72, 1:1,000); CyclinE1 (Abcam, ab3927, 1:1,000); CyclinE1 (Abcam, ab3927, 1:1,000); CyclinD1(Santa Cruz Biotechnology, sc-246, 1:1,000); β-actin (Abcam, ab15265, 1:1,000).

### siRNA and Plasmid Transfection

The siRNAs targeting MNX1 and p21^cip1^ were conducted and purchased from RiboBio, Guangzhou, China. All siRNA sequences are shown in Table S3. The full-length cDNA of human MNX1 were PCR-amplified and cloned into the expression vector Pgpu6/gfp/neo (Vigene Biosciences, Shandong, China). The siRNAs and overexpression plasmids were transfected into cervical cancer cells according to the Lipofectamine 3000 Reagent (Invitrogen, Carlsbad, CA, USA) protocol. Non-sense RNAi (si-NC) and empty plasmids (oe-NC) was used as negative controls.

### Cell Proliferation Assay

The cell proliferation assays were performed 24 h after transfection. For Real TimexCELLigence analysis system (RTCA), 8,000 cells/100 μL were seeded in E-plates, and the plates were locked into the RTCA DP device in the incubator to calculate the “cell index” value. In colony formation assay, a total of 200 cells were placed in afresh 6-well-plate and the cells were stained with 0.1% crystal violet solution after 10–14 days. For 5-ethynyl-2′-deoxyuridine (EdU) assay (keyFluor488 Click-IT EDU Kit, RiboBio, Guangzhou, China), the transfected cells were placed in 96-well-plates (8,000 cells/well) overnight in a CO_2_ incubator. Then, cells were incubated with 100 μL/well of 10 μM EdU for 2 h at 37°C and fixed with 50 μL 4% paraformaldehyde-containing PBS for 30 min at room temperature. Subsequently, the cells were cultured for 5 min with 50 μL of 2 mg/mL glycine and then washed with 100 μL 3% BSA in PBS. After permeabilization with 0.5% Triton X-100 for 20 min, the cells were cultured with 1 × Click-iT reaction solution for 30 min at room temperature in dark conditions. After that, cells were incubated with 100 μL/well of 1 × Hoechst 33,342 solutions for 30 min at room temperature in the dark after washing with 100 μL of PBS. The cells were then imaged using fluorescence microscopy and proliferation cell ratios were counted from five random fields in every well. Each experiment was repeated three times. A total of 400 cells in a fresh six-well-plates were maintained in medium containing 10% FBS; the medium was replaced every 3 or 4 days. After 2 weeks, the cells were fixed with 4% paraformaldehyde and stained with 0.1% crystal violet. Each experiment was repeated three times.

### Migration and Invasion Assay

For wound healing assay, cells were growing on the 6-well-plate, then artificial scratch on a confluent monolayer of cells was created with a 200 μL pipette tip. The medium was replaced with the serum-free and cells imaged 48 h later. For transwell and matrigel assay, totally 40,000 transfected cells were added to the upper chamber of Transwell assay inserts (8 μM PET, 24-well Millicell) or a Matrigel coated membrane (BD Biosciences) containing 200 μL serum-free DMEM media. The lower chambers were filled with 800 μl DMEM media containing 10% FBS. After a 24-h (migration assay) or 48-h (invasion assay) incubation, the cells were fixed with 4% polyformaldehyde, stained with crystal violet, and imaged. Migration and invasion were assessed by counting cell nuclei from five random fields on every filter. Each experiment was repeated three times. RTCA was also used to evaluated the ability of migration and invasion. CIM-plates installation and baseline measurement was carried out according to the instructions. Add 100 μl of mixed, serum-free cell suspension (4 × 10^4^ cells) to the upper chamber in CIM-plates, and the plates were locked into the RTCA DP device in the incubator to calculate the “cell index” value.

### Cell Cycle Analysis

Cells were digested with 0.25% trypsin-EDTA and fixed with 70% ethanol for 12 h at 4°C. The ethanol-suspended cells were centrifuged and stained with PI staining solution for 10 min in the dark at 37°C. A FACSCalibur flow cytometer was used to detect cell cycle distribution. The percentage of the cells in G0–G1, S, and G2–M were counted and compared.

### Chromatin Immunoprecipitation (ChIP)

Cells were cross-linked in 4% paraformaldehyde and the reaction was quenched with glycine. After two washes with cold PBS, cells were added with pre-cooling PBS containing cocktail (Halt™ Protease Inhibitor Cocktail, Thermo Scientific, #78430) and scraped into a centrifuge tube. The cells were centrifuged for 10 min at 800 g at 4°C, then added with 500 μL cell lysis buffer (containing 2.5 μL cocktail) and incubated on ice for 15 min. Cells were then centrifuged for 5 min at 800 × g, 4°C and cell precipitates were resuspended in 500 μL nucleus lysis buffer (containing 2.5 μL cocktail). The cells were sonicated (amplitude 30%) on ice for 10 min and soluble chromatin was obtained by centrifuging for 10 min at 12,000 g at 4°C. Five micrograms of anti-MNX1 antibody (Sigma-Aldrich; SAB2101494) coupled to magnetic beads (Resin M2, Sigma, Shanghai, China) was used to immunoprecipitate the DNA-protein complex, and the IgG antibody was used as a negative control. The immunoprecipitation products were washed with 500 μL low salt buffer, high salt buffer, LiCi buffer, and TE buffer successively, all for 5 min at 4°C. The ChIP elution buffer (containing proteinase K) was used for DNA purification. The beads were wiped out on a magnetic frame and the DNA was eluted with elution buffer C from adsorption column. ChIP DNA samples were subjected to PCR amplification with primers specific to p21^cip1^ promoter region. PCR products were then used for agarose gel electrophoresis. The sequence of primers used are shown in Table S4 and GAPDH was used as a control.

### Luciferase Reporter Assay

The p21^cip1^ (CDKN1A) promoter region (−2,000 bp) was amplified and cloned into luciferase reporter plasmid (pGL3-basic). The p21^cip1^ promoter wild-type plasmids or mutant-type plasmids were co-transfected with CMV-MNX1 expression plasmids in HEK293T cells, and CMV-empty vectors were used as a negative control. Relative luciferase activity was corrected for Renilla luciferase activity of pGL3-basic, and normalized to the activity of the control.

### Xenograft Model

All animal studies were conducted in accordance with NIH animal use guidelines and protocols were approved by Nanjing Medical University Animal Care Committee. Sixteen female nude mice (4–6 weeks old) were purchased from Nanjing Medical University School of Medicine's accredited animal facility. The mice were randomly divided into two groups using random number generator. In each group, 1.0 × 10^6^ exponentially growing cervical cancer cells were injected in axilla subcutaneously. Before tumor transplantation, cells were transfected with shRNAs or overexpression plasmids. The transfection was performed by transient transfection according to the specification of Lipofectamine 3000 (Invitrogen, Carlsbad, CA, USA). The sh-NC and empty vector pcDNA3.1 were used as controls and totally 5 μg plasmid vectors were transfected into cells for each group. The sequences of shRNAs are shown in Table S5. Tumors were harvested at 6 weeks after injection. The weight of tumor was measured on the scale and tumor volume was estimated using calipers ([length × width^2^]/2). And tissues were immunohistochemically stained with MNX1 (Abcam, ab79541, 1:100), Ki67 (Abcam, ab79541, 1:100), and p21^cip1^ (Abcam, ab109520, 1:100). Western blotting was performed as previously described. No blinding was done in the animal studies.

### Statistical Analysis

Results are presented as the mean ± standard deviation (SD). Statistical analyses were performed using SPSS Statistics (version 20.0, Chicago, Ill) and GraphPad Prism 6 software (GraphPad Software, Inc., La Jolla, CA, USA). *P* < 0.05 was considered statistically significant.

## Results

### Overexpression of MNX1 Correlates With Poorer Prognosis and More Aggressive Clinical Features

**Figure 1:**
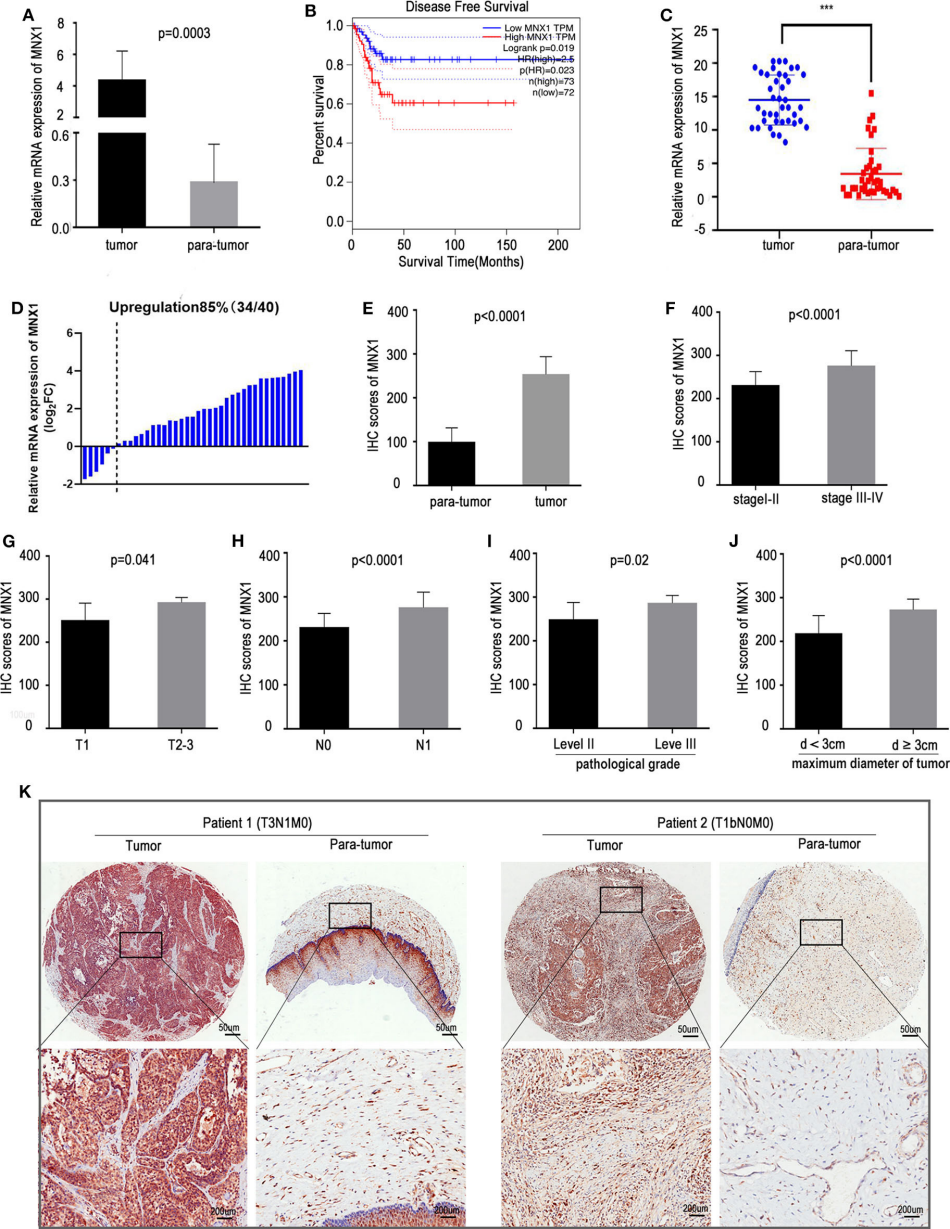
MNX1 is upregulated in CC tissues and positively correlates with aggressive clinical characteristics. **(A)** MNX1 is up-regulated in CC tissues compared with adjacent normal tissues in TCGA dataset (*P* = 0.0003). **(B)** Patients with high expression of MNX1 have poor Disease Free Survival (DFS) in CC (*p* = 0.023). **(C,D)** The mRNA expression of MNX1 in cervical cancer tissues was significantly higher than that in adjacent normal tissues in 85% (34/40) patients (*p* < 0.001). **(E)** The MNX1 staining score was up-regulated compared with that in adjacent normal tissues (*p* < 0.0001). **(F)** The MNX1 staining score was positively correlated with TNM stage (*p* < 0.0001), **(G)** T stage (*p* = 0.041), **(H)** lymph node metastasis (*p* < 0.0001), **(I)** tumor differentiation (*p* = 0.02), and **(J)** local primary tumor diameter (*p* < 0.0001) in CC patients. **(K)** Representative IHC staining images in TMAs were shown. Error bars represent the mean ± SD values. NS, No significance. *** represents *P* < 0.001.

Analysis of TCGA dataset revealed that the mRNA expression of MNX1 was remarkably upregulated in cervical cancer tissues compared with para-tumor tissues (*p* = 0.0003, Figure 1A). In GEPIA (Gene Expression Profiling Interactive Analysis) website, patients with higher expression of MNX1 bore a worse disease free survival (*n*_high_ = 73, *n*_low_ = 72, *p* = 0.019, Figure 1B). The expression of MNX1 in cervical cancer tissues were significantly higher than adjacent tissues in 85% (34/40) of 40 cervical cancer patients (*p* < 0.001, Figures 1C,D). IHC assays based on tissue microarrays (TMAs) were performed to detect the protein expression of MNX1 in 62 paired human cervical cancer tissues and para-tumor tissues, and results showed that staining scores of MNX1 were higher in cancer tissues (*p* < 0.0001, Figure 1E). Combined with the patients' clinical information, the expression of MNX1 was higher in patients with more advanced TNM stage (stage I–II vs. III–IV, *p* < 0.0001, Figure 1F), T stage (T1 vs. T2–T3, *p* = 0.041, Figure 1G), and N stage (N0 vs. N1, *p* < 0.0001, Figure 1H). Moreover, MNX1 staining scores were linked to higher pathological grade (level II vs. III, *p* = 0.02, Figure 1I) and larger tumor maximum diameter (*d* < 3 vs. ≥3 cm, *p* < 0.0001, Figure 1J). And IHC images of two patients with different clinical stages were presented (Figure 1K).

### Knockdown of MNX1 Inhibited Progression of Cervical Cancer *in vitro*

**Figure 2:**
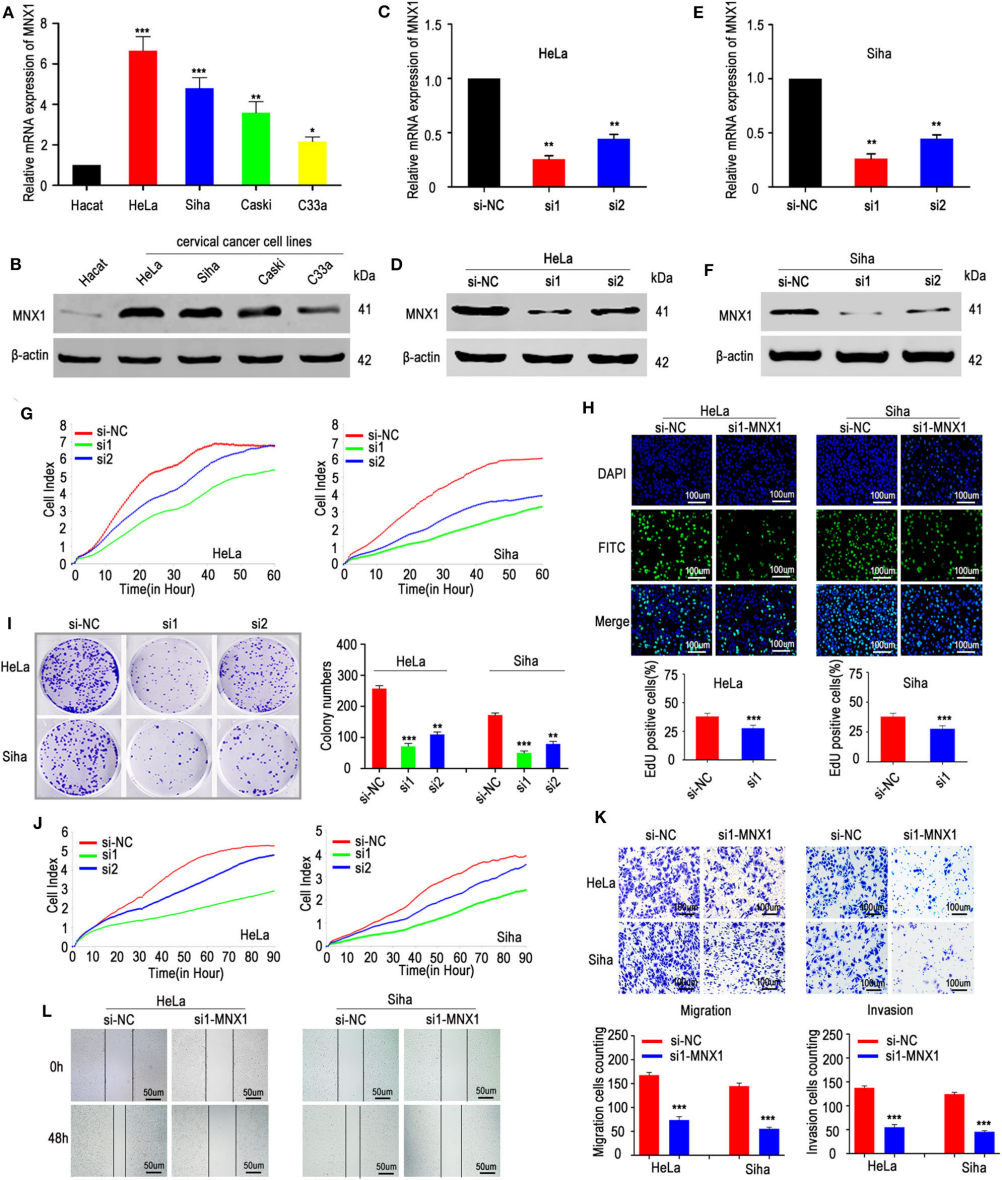
Knockdown of MNX1 suppressed the proliferation, migration, and invasion in CC cells. **(A,B)** MNX1 mRNA and protein level are upregulated in CC cell lines. **(C–F)** Two specific siRNA (si1 and si2) of MNX1 were designed and the transfection efficiencies of siRNAs in HeLa and Siha cells were analyzed by qRT-PCR and western blot. **(G–I)** The proliferation abilities were evaluated by xCELLigence system assay, EdU incorporation assay, and colony formation assay were inhibited after knockdown of MNX1 in HeLa and Siha cells. **(J)** The xCELLigence system assay, **(K)** Transwell and Matrigel assay, and **(L)** wound healing assay indicated that migration and invasion capacities were suppressed after si-MNX1 in HeLa and Siha cells. Error bars represent the mean ± SD values of three independent experiments. *P < 0.05, **P < 0.01, ***P < 0.001, NS, No significance.

To evaluate the expression of MNX1 in cell lines, qRT-PCR and western blotting were performed and results showed that MNX1 was generally upregulated in cervical cancer cell lines compared with normal human cervical cell lines (Hacat) (Figures 2A,B). To further investigate the biological function of MNX1 in cervical cancer, two specific siRNAs targeting MNX1 were transfected into HeLa and Siha cells. Both two siRNAs showed favorable suppression efficiency in HeLa (Figures 2C,D) and Siha cells (Figures 2E,F). The RTCA proliferation assay (Figure 2G), EDU assay (Figure 2H), and colony formation assay (Figure 2I) showed that knockdown of MNX1 inhibited the proliferation ability of cervical cancer in HeLa and Siha cells. Moreover, RTCA migration assay (Figure 2J), transwell assay, and matrigel assay (Figure 2K), and wound healing assay (Figure 2L) revealed that silencing MNX1 inhibited the ability of cervical cancer cells to migrate and invade. These results suggest that MNX1 plays a vital role in the malignant phenotype of cervical cancer.

### Ectopic Expression of MNX1 Enhanced Aggressiveness of Cervical Cancer *in vitro*

**Figure 3:**
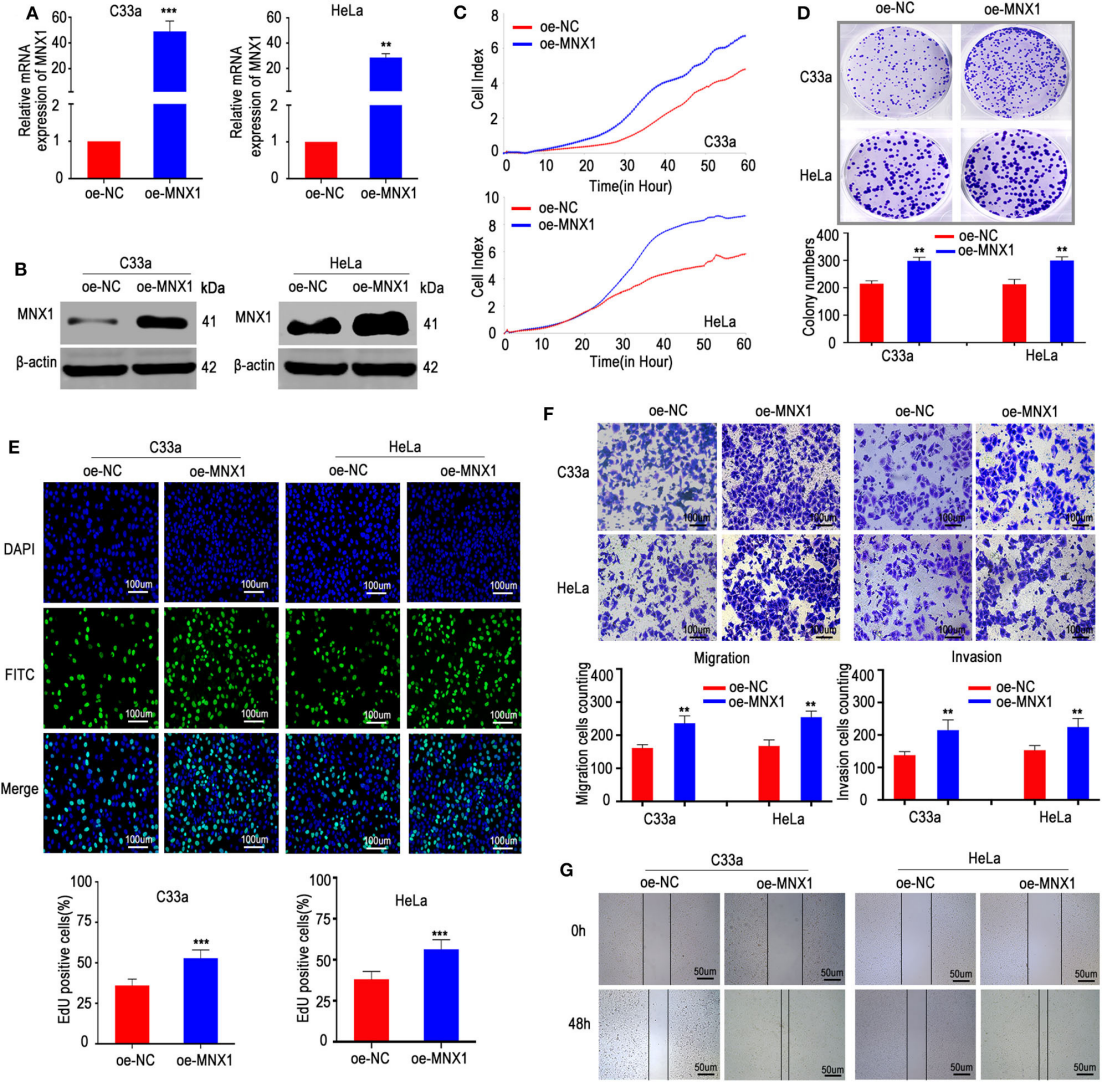
Ectopic expression of MNX1 enhanced aggressive abilities in C33a and HeLa cells. **(A,B)** The pcDNA3.1-MNX1 was synthesize and the transfection efficiencies were analyzed by qRT-PCR and western blot. The proliferation functions were measured by **(C)** the xCELLigence system assay, **(D)** colony formation assays, and **(E)** EdU incorporation assays were elevated in oe-MNX1 C33a and HeLa cells. **(F)** The Transwell assay and Matrigel invasion assay, **(G)** wound healing assay also showed that oe-MNX1 strengthened migration and invasion capacities. Error bars represent the mean ± SD values of three independent experiments. *P < 0.05, **P < 0.01, ***P < 0.001, NS, No significance.

To further verify the biological role of MNX1 in cervical cancer, a pcDNA3.1 plasmid to overexpress MNX1 was constructed and transfected into C33a and HeLa cells. The plasmid effectively upregulated the expression of MNX1, confirmed by qRT-PCR and western blotting (Figures 3A,B). Consistently, our results showed that ectopic expression of MNX1 promotes proliferation, migration, and invasion (Figures 3C–G) of cervical cancer cells.

### si-MNX1 Induced G2/M Cell Cycle Arrest and Upregulated the Expression of p21^cip1^

**Figure 4:**
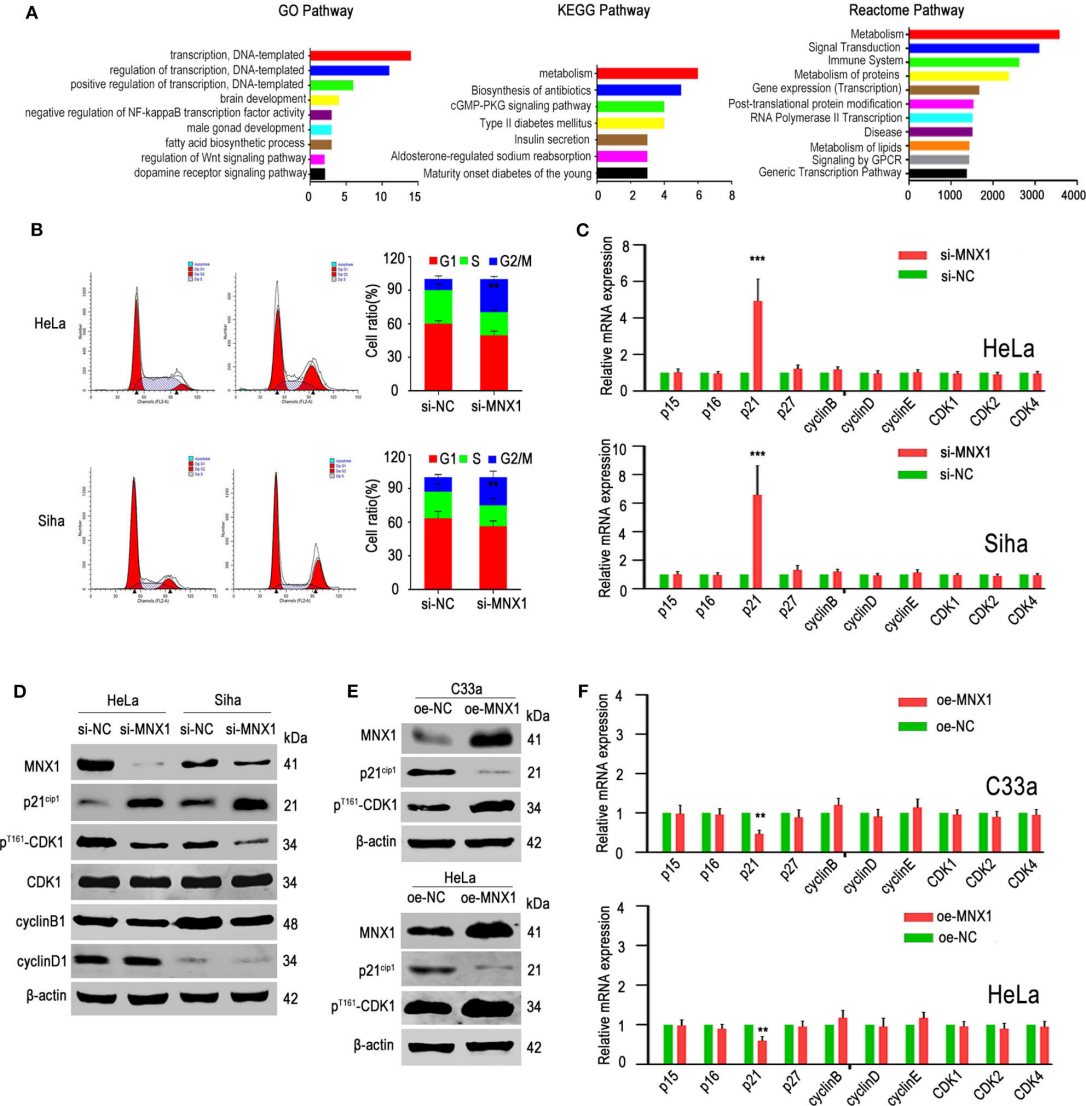
Knockdown of MNX1 expression induced G2/M phage arrest by regulating the p21^cip1^ expression. **(A)** Many genes were enriched in regulation of transcription by GO analysis. Most of the genes were enriched in the metabolic pathways by KEGG and Reactome pathway analysis. **(B)** Knockdown of MNX1 generated G2/M stage arrest in HeLa and Siha cells were measured by flow cytometry. **(C,F)** The p21^cip1^ mRNA levels were upregulated or downregulated after si- or oe-MNX1 in CC cell lines. **(D,E)** The protein level of p21^cip1^ was upregulated or downregulated while the expression of p^Thr161^-CDK1 was decreased or increased after knockdown or ectopic MNX1 of CC cells. The expression of CDK1, CCNE1, CCND1, and CCNB1 had no obvious changes. Error bars represent the mean ± SD values of three independent experiments. *P < 0.05, **P < 0.01, ***P < 0.001, NS, No significance.

Two hundred and eight genes highly correlated with MNX1 were used for GO, KEGG, and Reactome pathway analysis. Results showed that MNX1 may participate in “transcription” and “metabolism” pathway (Figure 4A). Cell cycle detection showed that knockdown of MNX1 induced G2/M cell cycle arrest in HeLa and Siha cells (Figure 4B). Furthermore, we examined the effect of MNX1 on the expression of cell cycle key-related genes, including p15^ink4b^, p16^ink4a^, p21^cip1^, p27^kip1^, CDK1, CDK2, CDK4, cyclinB1, cyclinD1, and cyclinE1. Both in HeLa and Siha cells, knockdown of MNX1 upregulated the expression of p21^cip1^, which has been confirmed as a tumor suppressor gene in multiple cancers (Figure 4C). And western blotting results suggested that knockdown of MNX1 increased the expression of p21^cip1^ while decreased the expression of phosphorylated CDK1 (p^Thr161^-CDK1), a downstream effector of p21^cip1^ (Figure 4D). Consistently with these results, ectopic expression of MNX1 decreased the expression of p21^cip1^ while increased the expression of p^Thr161^-CDK1 in C33a and HeLa cells (Figures 4E,F). It suggested that MNX1 might exerted its biological function via modulating the expression of p21^cip1^.

### MNX1 Suppressed the Expression of p21^cip1^ via Binding to Its Promoter Region

**Figure 5:**
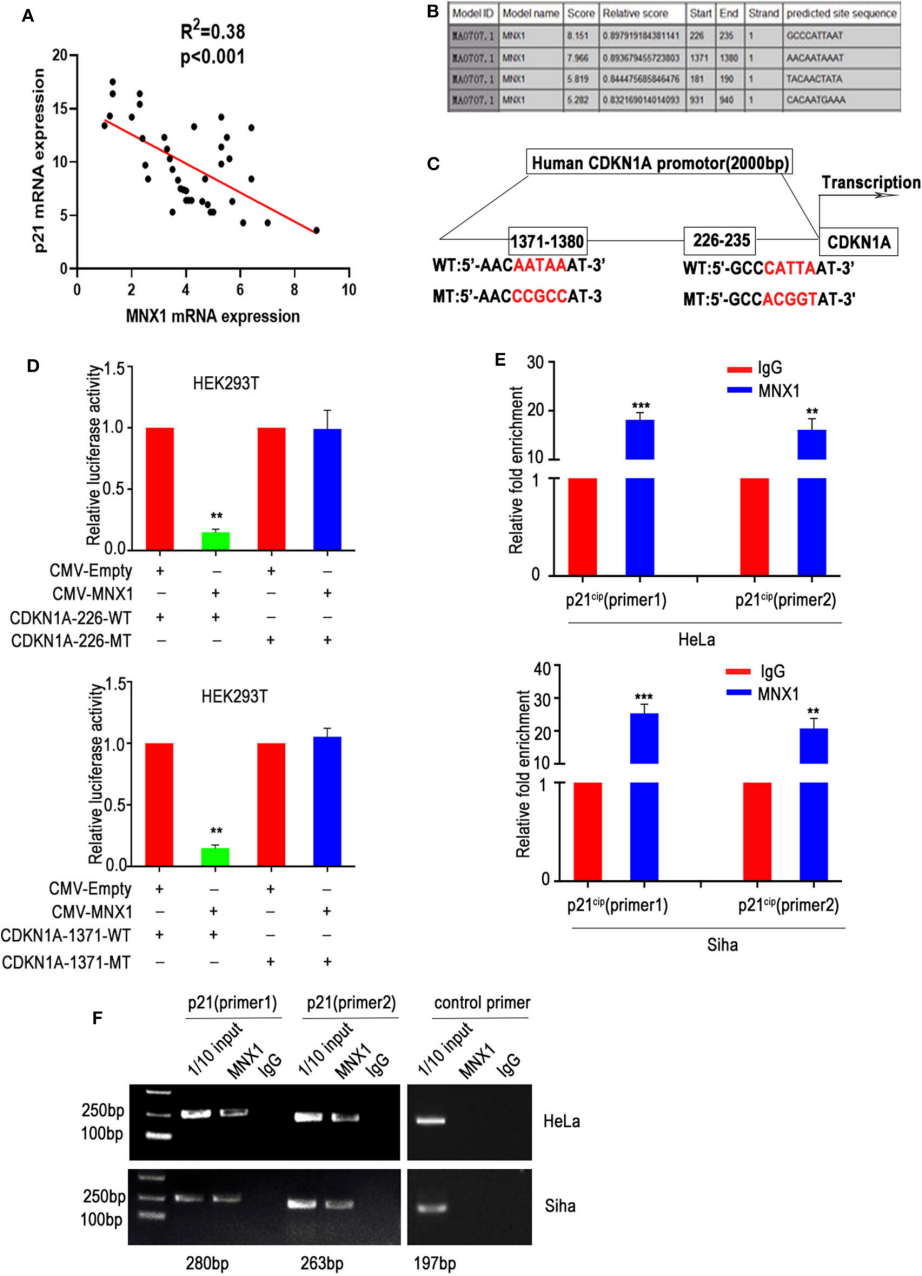
MNX1 bounds to the p21^cip1^ promoter region and suppresses p21^cip1^ transcription. **(A)** The expression of MNX1 and p21^cip1^ is negatively correlated in 40 cervical cancer tissues (*P* < 0.001). **(B)** The JARSPAR database indicates that MNX1 has several binding sites with the promoter region of p21^cip1^. **(C)** Schematic diagram shows that the two sites with the highest score of MNX1 on p21^cip1^ promoter and the mutant p21^cip1^ promoter were selected. **(D)** Overexpression of MNX1 remarkably decreased wild type but not mutant p21^cip1^ promoter luciferase activity (p21^cip1^-226, *p* < 0.01; p21^cip1^-1371, *p* < 0.01). **(E)** Chromatin immunoprecipitation (ChIP) assays using normal IgG or anti-MNX1 demonstrated that MNX1 directly binding to p21^cip1^ promoter region. **(F)** The results of ChIP-PCR product electrophoresis were showed that a clear band was observed in the anti-MNX1 group, while almost no band was detected in the IgG control group. **P < 0.01, ***P < 0.001.

Our previous results showed that knockdown or ectopic expression of MNX1 altered the expression of p21^cip1^. To further verify the mechanism, we analyzed the correlation between MNX1 and p21^cip1^ in 40 cases of CC samples, and the results were shown that MNX1 and p21^cip1^ had a negative correlation (*n* = 40, *p* < 0.001) (Figure 5A). As transcription factors usually bind to sequence-specific DNA to regulate transcription, we utilized Jaspar Database to predict the binding site between MNX1 and the promoter region (upstream 2,000 bp of coding region) of CDKN1A (the gene symbol of p21^cip1^). It turned out that MNX1 was predicted to have four binding sites with the promoter region of CDKN1A, of which 1,371–1,380 bp (AACAATAAAT) and 226–235 bp (GCCCATTAAT) showed higher combination scores (Figure 5B). Accordingly, the wild CDKN1A promoter region and mutant types (226-MT and 1371-MT) were generated and cloned into luciferase reporter vector (pGL3-basic; Figure 5C). And in luciferase reporter assay, overexpression of MNX1 inhibited the transcriptional activity of the wild CDKN1A promoter but not mutant type (Figure 5D). Moreover, ChIP assay also revealed that MNX1 bound to the p21^cip1^ promoter region in HeLa and Siha cells (Figures 5E,F).

### Silencing p21^cip1^ Rescued the Function of si-MNX1

**Figure 6:**
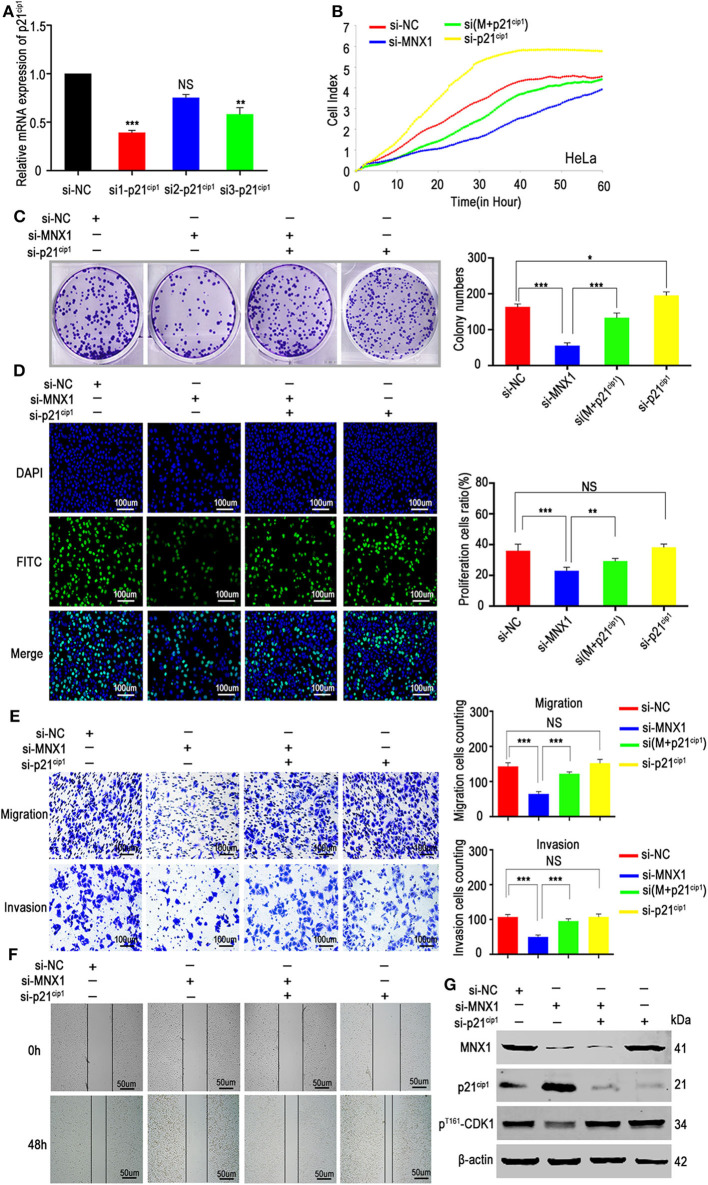
Downregulation of p21^cip1^ partially recovered the malignant phenotypes of si-MNX1 cells. **(A)** The transfection efficiency of p21^cip1^ was determined by qRT-PCR and si1-p21^cip1^ was chosen to further experiments. **(B–D)** The proliferative abilities were partially rescued after knockdown p21^cip1^ in si-MNX1 HeLa cells were measured by the xCELLigence system assay, colony formation assay, and EdU incorporation assay. **(E,F)** The invasion and migration capacities have also been significantly improved after knockdown p21^cip1^ in si-MNX1 cells compared with si-MNX1 alone cells. **(G)** The protein level of p21^cip1^ and p^Thr161^-CDK1 were partially reversed when knockdown of p21^cip1^ in si-MNX1 compared with si-MNX1 alone. Error bars represent the mean ± SD values of three independent experiments. *P < 0.05, **P < 0.01, ***P < 0.001, NS, No significance.

To determine whether the function of MNX1 was relied on p21^cip1^, we designed three siRNAs (Table S3) to knockdown the expression of p21^cip1^ in HeLa cells. The si1-p21^cip1^ showed the best transfection efficiency (Figure 6A) and it was used for the following experiment. RTCA proliferation assay, colony formation assay, EDU assay, transwell assay, matrigel assay, and would healing assay revealed that silencing p21^cip1^ partially rescued the decreased proliferation, migration, and invasion ability of HeLa cells caused by knockdown of MNX1 (Figures 6B–F). And western blotting showed that the protein level of p21^cip1^ and p^Thr161^-CDK1 were partially reversed by silencing p21^cip1^ (Figure 6G).

### MNX1 Promoted Tumor Growth of Cervical Cancer *in vivo*

**Figure 7:**
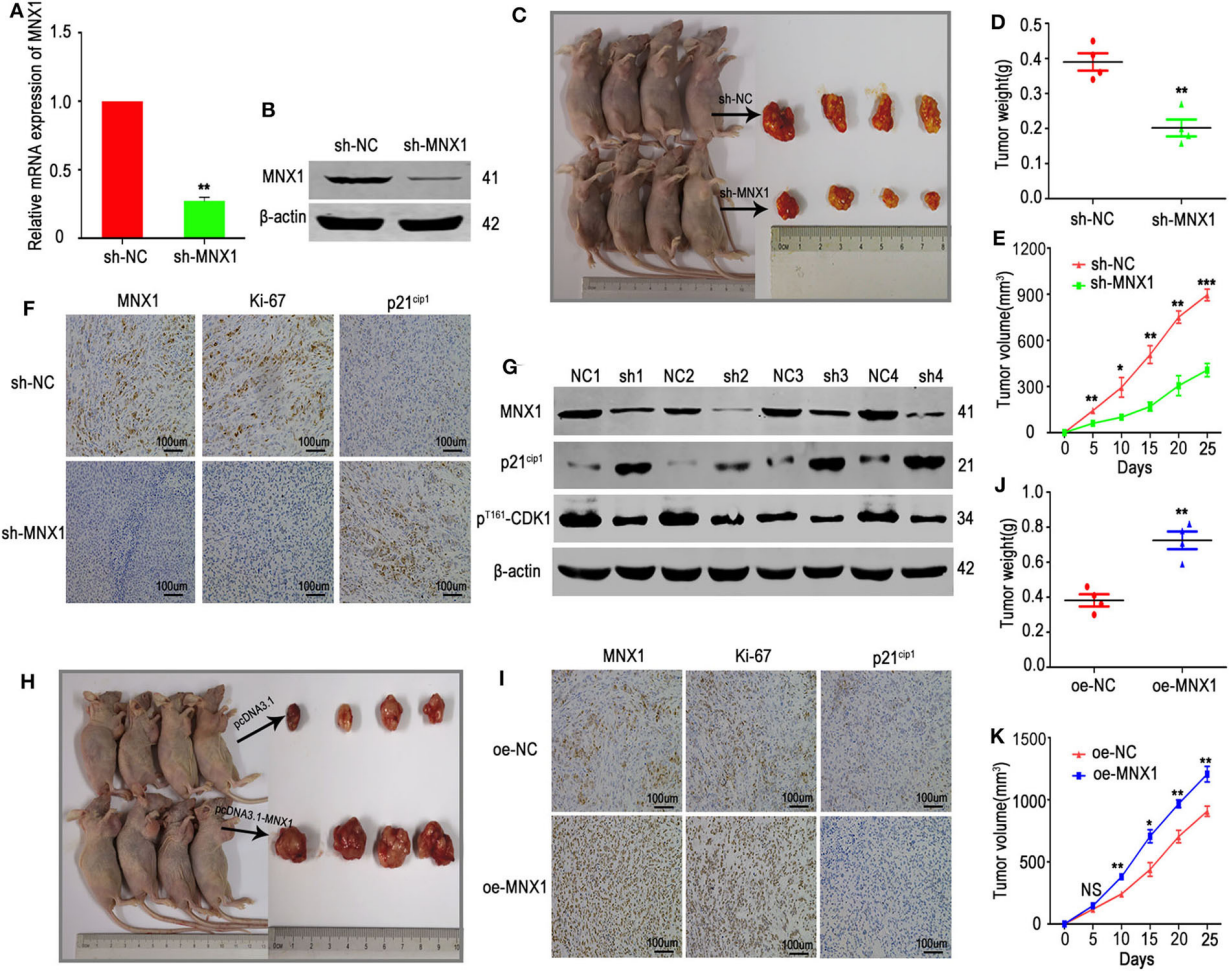
Knockdown or overexpression of MNX1 inhibited or promoted tumor growth *in vivo*. **(A,B)** The transfection efficiency of sh-MNX1 was measured by qRT-PCR and Western blot. **(C)** A total of eight nude female mice were sacrificed and xenograft tumors were collected after injection with sh-MNX1 cells 6 weeks. **(D,E)** Tumor volume and weight were reduced in the sh-MNX1 group compared with those in the sh-NC group. **(F)** The expression of MNX1 and Ki-67 was downregulated and p21^cip1^ was upregulated in sh-MNX1 xenograft tumors analyzing by IHC staining. **(G)** The protein level of MNX1, p^Thr161^-CDK1 were downregulated and p21^cip1^ was upregulated in sh-MNX1 mouse xenograft tumors analyzed by western blot. **(H)** A total of eight nude female mice were sacrificed and xenograft tumors were collected after injection with oe-MNX1 cells 6 weeks. **(J,K)** Tumor volume and weight was increased in the oe-MNX1 group compared with those in the oe-NC group. **(I)** The expression of MNX1 and Ki-67 was upregulated and p21^cip1^ was downregulated in oe-MNX1 xenograft tumors analyzing by IHC staining. Error bars represent the mean ± SD values. *P < 0.05, **P < 0.01, ***P < 0.001, NS, No significance.

The xenograft models were used to explore the function of MNX1 *in vivo*. The shRNA-MNX1 (shRNA-NC as control) was transfected into HeLa cells and the knockdown efficiency was confirmed by qRT-PCR and western blotting (Figures 7A,B). Results showed that knockdown of MNX1 inhibited tumor growth (measured by tumor weight and volume) *in vivo* (Figures 7C–E). IHC staining and western blotting of harvested tumors revealed that knockdown of MNX1 upregulated the protein level of p21^cip1^ and downregulated ki-67 and p^Thr161^-CDK1 *in vivo* (Figures 7F,G). Moreover, ectopic expression of MNX1 promoted tumor growth and altered the expression of p21^cip1^ and ki-67 *in vivo* (Figures 7H–K).

## Discussion

In this study, we identified MNX1, a transcription factor of homeobox family, was significantly upregulated and involved in the progression of cervical cancer. The overexpression of MNX1 correlated with advanced clinical stages and poorer prognosis of cervical cancer patients. Furthermore, MNX1 exerted its oncogenic role via modulating the expression of p21^cip1^, especially by targeting the promoter region of p21^cip1^ thus to repress its transcription. In accordance with our findings, a recent article showed that MNX1 had a role in the progression of cervical cancer, partially through upregulating cell cycle regulators CCNE1 and CCNE2 (24). And MNX-AS1, the antisense lncRNA of MNX1, was also reported to promote the invasion and metastasis of gastric cancer through repression of CDKN1A (25). All this results indicated that MNX1 played a critical role in cancer growth and cell cycle progression, and MNX1 might serve as a useful diagnostic and treatment target for cervical cancer.

MNX1is a member of homeobox gene family, which all contain a homeobox (a DNA sequence, around 180 base pairs long) and encode homeodomain protein products as transcription factors (26). This cluster of genes has been identified to participate in the regulation of development and morphogenesis in animals, fungi, and plants (27). For example, CDX1, which is stably expressed in the human intestine, plays an important role in embryonic epicardial development (28, 29). And the protagonist of our study, MNX1, participates in motor neuron development and neurodegenerative processes of zebrafish (30) and moreover controls cell fate choice in the developing endocrine pancreas (31). In recent years, more and more researches uncovered the role of development-related homeobox genes in carcinogenesis and these genes show great application prospect in tumor diagnosis and prevention, as the role of carcino-embryonic antigen (CEA) in gastroenteric tumors and alpha fetal protein (AFP) in liver cancer (32–34). For instance, PDX1 is a key regulator in pancreatic development and β-cell function (35) and meanwhile dynamically regulates pancreatic ductal adenocarcinoma initiation and maintenance (36). HOXC13, a highly conserved transcription factor involved in morphogenesis of all multicellular organisms, is aberrantly expressed and associated with cancer progression in esophageal cancer (37), lung adenocarcinoma (38), and liposarcomas (39). Likewise, MNX1 has been reported to promote sustained proliferation in bladder cancer by upregulating CCNE1/2 (40) and to act as a novel oncogene in prostate cancer (41). And in our study, MNX1 was also confirmed to be upregulated in cervical cancer and enhance the progression of cervical cancer.

In terms of mechanism, we found that MNX1 promoted tumor growth of cervical cancer via accelerating the progression of the cell cycle, especially by modulating the expression of p21^cip1^. Cell cycle is a vital process by which a cell leads to duplication and disorders of the cell cycle regulation may lead to tumor formation (42). The cell cycle progress is determined by two types of regulatory factors, cyclins and cyclin-dependent kinases (CDKs) (43). Active cyclin-CDK complexes phosphorylate proteins to elevate the expression levels of cyclins and enzymes required for DNA replication (44). Conversely, the cell cycle progression can be prevented by inhibitors by binding to and thus inactivating cyclin-CDK complexes, such as p21^cip1^, p27^kip1^, p16^ink4a^, and so on (45). The p21^cip1^, also known as cyclin-dependent kinase inhibitor 1 (CDKN1A), has been identified as a regulator of cell cycle and a tumor suppressor in multiple kinds of cancers (46). Our results proved that MNX1 repressed the transcription of p21^cip1^ by directly targeting its promoter region and furthermore promoted the phosphorylation of downstream CDK1. The MNX1-p21^cip1^-p^Thr161^CDK1 axis played crucial roles in the progression of cervical cancer and meanwhile provided new evidence for the pathogenesis of cervical cancer. Moreover, the association between cervical cancer and HPV has long been identified. As a sexually transmitted agent, HPV are involved in transformation and maintaining of transformed status (47). Many studies have reported that HPV can also alter the expression of p21 (48–50). Thus, we searched the GEO dataset to seek for information about the relationship between MNX1 and HPV viral infection. We analyzed the GSE dataset 103,546 and found that there were no significant changes in the expression of MNX1 (NM_005515) in HaCat cells infected with HPV11E6 or HPV18E6. In GSE3292 (GDS1667), HPV positive or negative head and neck squamous cell carcinoma (HNSCC) showed no expression differences of MNX1 (Figure S1). This information suggests that MNX1 might not be directly involved in HPV carcinogenesis and further investigations are needed in the future. In addition, cervical cancer is almost invariably associated with p53 loss (either mutation of HPV infection) and p53 is a very well know activator of p21. In this study, we proved that MNX1 exerted an oncogenic role in cervical cancer via suppressing the expression of p21 with binding to its promoter region. And we performed qRT-PCR and western blot in cervical cancer cells and results showed that knockdown of MNX1 did not affect the expression of p53. Transcription factor could directly regulate the expression of target genes by binding to the gene promoter. We speculated that MNX1 mediated downregulation of p21 was independent on p53. And in this study, three cervical cancer cells (HeLA, Siha, and C33A) were used to study the function of MNX1. We noted that p53 was lowly expressed in Hela cells, positively expressed in Siha cells and mutant of codon 273 in C33A cells. It indicated that MNX1 could function in cervical cancer cells with different p53 status. Moreover, it has been reported that in p53 mutation acute myeloid leukemia, MNX1 was identified as one of the hub genes from the protein–protein interaction network (51). And in hematopoietic stem and progenitor cells, the oncogenic potential of MNX1 was mediated via p53-p21 signaling pathway (52). In breast cancer, enrichment analysis suggested that MNX1 is probably involved in biological processes and pathways related to nuclear division, cell cycle, and p53 signaling (18). And a recent study showed that MNX1 had a role in the progression of cervical cancer, partially through upregulating cell cycle regulators CCNE1 and CCNE2 (24). These studies suggested that the function of MNX1 might be translatable to other tumor types.

In summary, we identified the homeobox member MNX1 as a tumor-promoting gene in cervical cancer. The upregulated MNX1 correlated with more advanced clinical pathological characteristics and poorer prognosis of cervical cancer patients. And MNX1 exerted its oncogenic role by repressing the transcription of p21^cip1^ thus to promote the progression of cell cycle. We believe that there are other genes beside p21 regulated by MNX1 in cervical cancer. RNA-seq and ChIP-seq experiments may need to confirm this cluster of genes. And as the downregulation of MNX1 inhibited tumor growth of cervical cancer, MNX1 may represent promising targets for the diagnosis and anti-tumor therapy in cervical cancer patients. Through knockdown of MNX1, it might combine the function of MNX1 with chemotherapy.

## Data Availability Statement

The original contributions presented in the study are included in the article/supplementary files, further inquiries can be directed to the corresponding author/s.

## Ethics Statement

The studies involving human participants were reviewed and approved by Ethics Committee of the Nanjing Medical University Affiliated Cancer Hospital. The patients/participants provided their written informed consent to participate in this study. The animal study was reviewed and approved by Nanjing Medical University Animal Care Committee.

## Author Contributions

LX, EL, and BR designed and supervised the study. BZ, YW, and JL were responsible for acquisition of data, interpretation of data, and article drafting. QZ contributed to experiments *in vitro*. JH and QL helped to analyze the data and revise the article. All authors contributed to the article and approved the submitted version.

## Conflict of Interest

The authors declare that the research was conducted in the absence of any commercial or financial relationships that could be construed as a potential conflict of interest.
