# Supplementary material for: MNX1 Promotes Malignant Progression of Cervical Cancer via Repressing the Transcription of p21cip1
Source: Front Oncol. 2020 Aug 11;10:1307. doi: 10.3389/fonc.2020.01307 (PMC7431913; doi:10.3389/fonc.2020.01307)
Supplement: Supplementary file 6 [file Image_1.pdf]

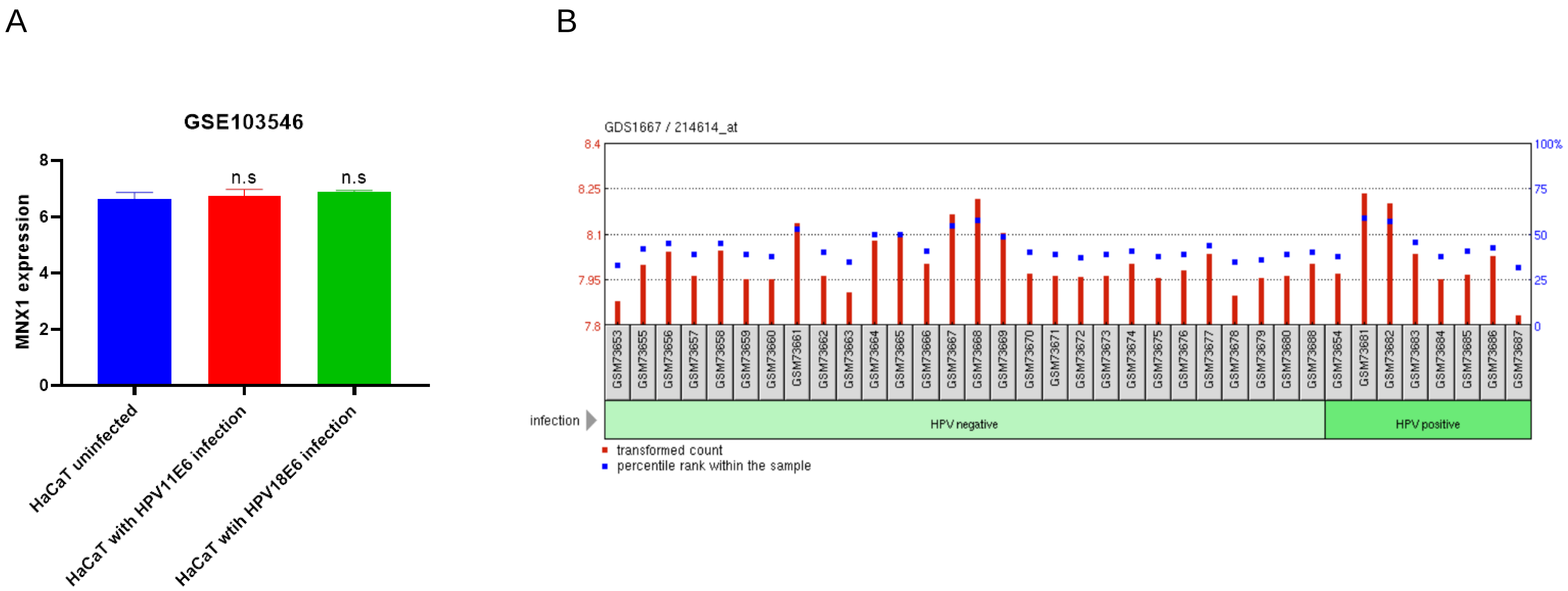

FigureS1: The relationship between MNX1 and HPV viral infection. A. In GSE103546, there were no significant changes in the expression of MNX1 (NM_005515) in HaCat cells infected with HPV11E6 or HPV18E6. B. In GSE3292 (GDS1667), head and neck squamous cell carcinoma (HNSCC) tumors with positive or negative of human papilloma virus (HPV) showed no expression differences of MNX1.
